# *Streptomyces calvus* Isolate 27 Promotes Plant Growth Through Hormone Accumulation and Bioactive Compounds

**DOI:** 10.3390/plants15091315

**Published:** 2026-04-25

**Authors:** Mayra Santiago-Velasco, Enrique González-Pérez, Raúl Rodríguez-Guerra, Alicia Becerra-Flora, Juan Francisco Jiménez-Bremont

**Affiliations:** 1 Laboratorio de Biotecnología Molecular de Plantas, División de Biología Molecular, Instituto Potosino de Investigación Científica y Tecnológica, A. C., Camino a la Presa de San José 2055, Lomas 4ta Sección, C.P., San Luis Potosí 78216, SLP, México; mayra.santiago@ipicyt.edu.mx (M.S.-V.); enrique.glez6891@gmail.com (E.G.-P.); abecerra@ipicyt.edu.mx (A.B.-F.); 2 INIFAP, Campo Experimental General Terán, Km. 31 Carretera Montemorelos-China, C.P., General Terán 67400, NL, México; raulrdzg@yahoo.com.mx

**Keywords:** *Arabidopsis thaliana*, phytohormones, plant–actinobacteria interactions, sustainable agriculture, volatile organic compounds

## Abstract

Some actinobacterial species have been reported to improve plant growth due to their roles as biostimulants and biological control agents. In this study, the effect of actinobacterial isolate 27, obtained from the rhizospheric soil of melon plants and identified as *Streptomyces calvus*, was evaluated on the growth of *Arabidopsis thaliana* and tomato plants. In *Arabidopsis*, *in vitro* assays showed that after seven days of interaction, isolate 27 increased fresh weight by 1.4-, 1.5-, and 2.3-fold and lateral root number by 1.7-, 1.3-, and 2.5-fold under physical contact and split-plate systems (MS and ISP2 media), respectively, compared with non-inoculated plants. An increased β-glucuronidase (GUS, encoded by the *uidA* gene) signal was observed in primary and lateral roots of the *Arabidopsis* DR5::*uidA* reporter line during both interaction types, suggesting the activation of auxin-responsive pathways. In addition, isolate 27 rescued the *rhd6* (*root hair defective 6*) mutant phenotype, restoring root hair formation. Gas chromatography-mass spectrometry (GC-MS) analysis revealed that isolate 27 emitted volatile organic compounds (VOCs), including an alcohol and several sesquiterpenes, and that this profile changed during interaction with *Arabidopsis* plantlets. In soil-based pot assays, inoculation with isolate 27 significantly enhanced the development of *Arabidopsis* plants after 23 days, both when applied alone and in co-inoculation with *Trichoderma atroviride*. Furthermore, isolate 27 stimulated tomato plant growth, leading to significant increases in fresh and dry biomass, as well as shoot and root lengths after 28 days. Overall, these results demonstrate that *S. calvus* isolate 27 promotes plant growth and development through the production of bioactive compounds that modulate plant growth pathways, including hormonal responses, highlighting its potential as a bioinoculant for sustainable and productive agricultural systems.

## 1. Introduction

Currently, agriculture faces increasingly complex challenges, including the impacts of climate change, the need to ensure global food security, and reduced crop yields associated with soil degradation and the proliferation of plant diseases [1,2]. These challenges are further compounded by the intensive use of agrochemicals, which not only reduces soil fertility but also generates negative environmental impacts. Consequently, efforts have focused on developing sustainable alternatives that maintain crop yields, ensure food security, and mitigate the adverse effects of agrochemical use [3]. The transition toward efficient agricultural systems based on environmental sustainability represents a key strategy to minimize agrochemical inputs and maximize crop productivity.

Plants establish symbiotic associations with a wide range of beneficial microorganisms, including bacteria and fungi. These interactions can enhance plant growth and development [4,5,6]. An increasingly adopted strategy in agriculture is the use of plant growth-promoting microorganisms (PGPM) as biofertilizers and biostimulants, which contribute to restoring soil fertility, improving plant health, and increasing crop productivity. These beneficial microorganisms stimulate plant growth by enhancing nutrient acquisition through increased bioavailability of organic and inorganic compounds [7,8]. In return, plants release root exudates that serve as carbon and energy sources for these microbes. This symbiotic interaction also promotes the production of secondary metabolites, contributing to plant development and improving tolerance to both biotic and abiotic stresses [9,10].

Plant growth-promoting bacteria encompass diverse phylogenetic groups, such as Firmicutes, Bacteroidetes, Proteobacteria, and Actinobacteria [11,12]. Among the most prominent genera within these phyla are *Bacillus*, *Pseudomonas*, *Rhizobium*, *Azospirillum*, and *Streptomyces*, which have been widely documented for their beneficial effects on plants [13]. Their association with plants is influenced by environmental conditions, plant genotype, and the specific type of plant-microorganism interaction established [14,15]. Within this group, Actinobacteria have attracted increasing attention due to their ecological and biotechnological importance [16]. They play key ecological roles, including the degradation of plant organic matter and the enhancement of nutrient uptake through mechanisms such as nitrogen fixation, phosphate and potassium solubilization, and the production of siderophores that improve iron availability [17,18]. These Gram-positive bacteria, characterized by a high G + C genomic content and large genomes, constitute a significant component of the soil microbiome [19,20]. In addition to their well-known ability to synthesize antibiotics, Actinobacteria exhibit remarkable metabolic versatility, enabling them to produce phytohormones, siderophores, and a wide range of bioactive compounds that directly promote plant growth and enhance tolerance to environmental stresses [17,19,21,22]. Moreover, many species, particularly those belonging to the genus *Streptomyces*, display a distinctive life cycle characterized by the formation of filamentous mycelia and spores. These structures play a key role in their survival, dispersal, and successful colonization of diverse environments, including the rhizosphere [20,23].

Many species of the genus *Streptomyces* produce the volatile compound geosmin, which contributes to the characteristic earthy odor of moist soil [21,24]. This genus is particularly notable for its abundance in soil and its ability to establish beneficial interactions with plants, functioning as both a growth promoter and a biocontrol agent. Their role in enhancing plant development has been documented in several crops, including tomato [25], sorghum, rice [26], and cucumber [27]. The formation of mycelia and spores not only facilitates dispersal and survival but also provides a competitive advantage by increasing resistance to prolonged periods of water scarcity and nutrient limitation [28].

The aim of this study was to evaluate the effects of the actinobacterial isolate 27, obtained from melon rhizospheric soil and identified as *Streptomyces calvus*, on the growth of *Arabidopsis thaliana* under *in vitro* conditions and in pot assays, as well as on tomato plants under pot conditions. Furthermore, it was examined whether *S. calvus* isolate 27 induces auxin accumulation using the DR5::*uidA* reporter line and whether it can restore root hair formation in the *rhd6* mutant. In addition, volatile organic compounds (VOCs) emitted by isolate 27 during its interaction with *A. thaliana* seedlings were identified. The effect of co-inoculation of *S. calvus* with the beneficial fungus *Trichoderma atroviride* on plant growth promotion was also evaluated. This study represents, according to current knowledge, the first report of *S. calvus* as a plant growth-promoting microorganism and highlights its potential as a promising biostimulant for sustainable agriculture.

## 2. Results

### 2.1. Identification and Characterization of Actinobacterial Isolate 27

Actinobacterial isolate 27, obtained from melon rhizospheric soil, was characterized. Initially, molecular identification was performed by amplifying the 16S rRNA gene, yielding a 1.3 kb fragment from genomic DNA (GenBank accession number PX674612). BLAST analysis of the obtained sequence using the NCBI database revealed 99.9% identity with *Streptomyces calvus* strains. A phylogenetic tree was constructed using the 16S rRNA gene sequence of isolate 27, along with other sequences of related species obtained from the NCBI database. The tree was rooted using *Nocardia casuarinae* as an outgroup, and the evolutionary relationships were inferred using the Neighbor-Joining method, as shown in Supplementary Figure S1. The phylogenetic analysis revealed that isolate 27 clusters within the *S. calvus* species clade, while the other clade included *S*. *djakartensis*, *S*. *tuirus*, *S*. *rochei*, and *S*. *mutabilis*.

**Figure 1:**
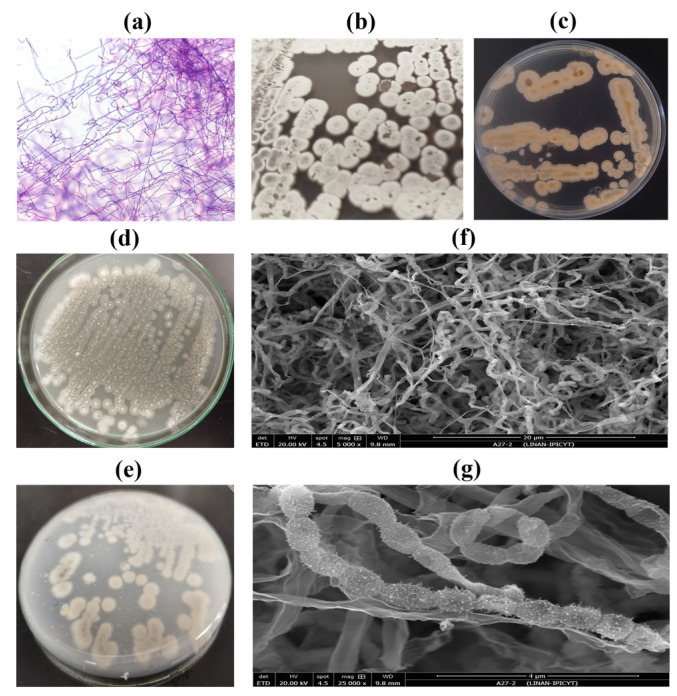
Morphology of *Streptomyces calvus* isolate 27. (**a**) Mycelium stained with crystal violet and observed under light microscopy (100× magnification). (**b**,**c**) Colony morphology after 14 days of growth on ISP2 medium. (**d**,**e**) Colony morphology after 14 days of growth on ISP3 medium. (**f**,**g**) Aerial mycelium and spore chains observed by scanning electron microscopy (SEM) after 14 days of growth on ISP2 medium.

Subsequently, morphological characterization of isolate 27 was performed based on previously reported characteristics of the species *S. calvus*. Isolate 27 showed Gram-positive staining, consistent with members of the phylum Actinobacteria, and branched filamentous structures characteristic of the genus *Streptomyces* were observed (Figure 1a). The colony morphology of *S. calvus* isolate 27 grown on ISP2 and ISP3 media is shown in Figure 1b and Figure 1d, respectively. After 14 days of incubation at 28 °C, growth on ISP2 medium resulted in well-developed aerial and substrate mycelia (Figure 1b). The aerial mycelium was white, whereas the substrate mycelium exhibited a sand-yellow coloration (Figure 1c). In contrast, growth on ISP3 medium revealed limited mycelial development, consistent with previous observations [29]. Under this condition, aerial mycelium formation was sparse and grayish (Figure 1d), while the substrate mycelium appeared whitish (Figure 1e). No diffusible pigments were detected on either ISP2 or ISP3 media, consistent with the findings previously reported [29]. Scanning electron micrographs of isolate 27 grown on ISP2 medium for 14 days (Figure 1f,g) revealed branched aerial hyphae, fragmented spore chains, and spores with spiny surfaces.

### 2.2. Effect of Streptomyces calvus Inoculation on the Growth of Arabidopsis thaliana Plantlets

**Figure 2:**
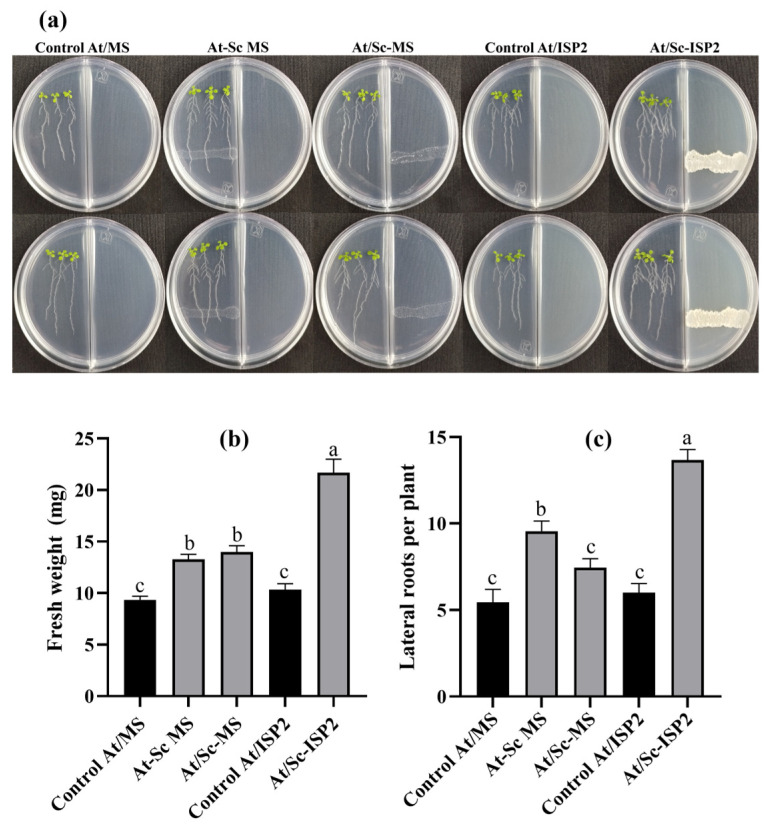
Growth-promoting effects of *Streptomyces calvus* isolate 27 on *Arabidopsis thaliana* seedlings. (**a**) Representative images of *A. thaliana* (At) plantlets grown on MS medium at 7 dpi with *S. calvus* isolate 27 under different experimental conditions: seedlings grown on MS without bacteria (control At/MS); direct interaction with the bacterium cultured on MS medium (At-Sc MS); split-plate interaction with bacterium cultured on MS medium (At/Sc-MS); seedlings grown on MS medium with ISP2 in the opposite compartment (control At/ISP2); and split-plate interaction with the bacterium cultured on ISP2 medium (At/Sc-ISP2). (**b**) Fresh weight obtained from each treatment (*n* = 6); (**c**) Number of lateral roots per plant (*n* = 18). Data are expressed as means ± SE. Different letters indicate statistically significant differences between treatments (one-way ANOVA followed by Dunnett’s test for comparisons against the control; *p* ≤ 0.05).

To evaluate the plant growth-promoting activity of *S. calvus* isolate 27, six-day-old *A. thaliana* Col-0 (At) seedlings were inoculated with the bacterium. The bacterial inoculum was prepared by culturing the isolate for two days at 28 °C before seedling inoculation. The growth-promoting effect of *S. calvus* isolate 27 was evaluated under both direct contact and split-plate interaction conditions to assess volatile-mediated effects (Figure 2a). For direct contact assays, the bacterium was grown on MS medium. For split-plate interaction assays, isolate 27 was cultivated on either MS medium or ISP2 medium, which is optimal for actinobacterial growth, in a divided plate configuration that prevented physical contact while allowing volatile exchange. Plant growth promotion was assessed 7 days post-inoculation (dpi) by measuring fresh weight and the number of lateral roots. Inoculation with *S. calvus* significantly increased fresh weight compared to uninoculated controls (Figure 2b). Although growth promotion was observed on MS medium under both interaction conditions, At-Sc MS (1.4-fold) and At/Sc-MS (1.5-fold), the highest fresh weight was obtained when the bacterium was grown on ISP2 medium (At/Sc-ISP2, 2.3-fold). Regarding root architecture, the At-Sc MS (1.7-fold) and At/Sc-ISP2 (2.5-fold) co-cultures induced the highest number of lateral roots, whereas At/Sc-MS showed only a slight increase (1.3-fold) and did not differ significantly from the MS control (Figure 2c). These results show that plant growth promotion is mediated by both diffusible non-volatile compounds and volatile organic compounds (VOCs). Notably, the effect was more pronounced when the bacterium was grown on ISP2 medium in the opposite compartment, leading to enhanced plant development.

### 2.3. S. calvus Isolate 27 Enhances Auxin Accumulation in Arabidopsis DR5::uidA Seedlings

**Figure 3:**
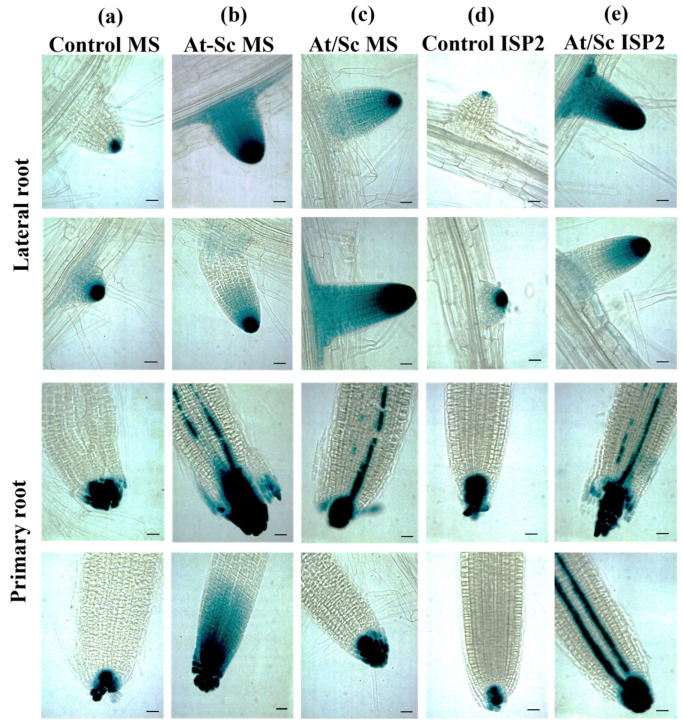
Effect of direct and split interactions with the actinobacterium *S. calvus* isolate 27 on auxin-responsive expression in the *Arabidopsis DR5::uidA* reporter line. Representative images of primary and lateral roots from *DR5::uidA* seedlings co-cultivated with *S. calvus.* Four-day-old seedlings were grown for 6 days under the following conditions: (**a**) Control (MS/MS), split plates containing MS in both compartments; (**b**) Direct interaction (At-Sc MS), seedlings in contact with the bacterium on MS; (**c**) Split interaction (At/Sc MS), seedlings on MS with the bacterium grown on MS in the opposite compartment; (**d**) Control (MS/ISP2), seedlings on MS facing sterile ISP2; and (**e**) Split interaction (At/Sc ISP2), seedlings on MS with the bacterium grown on ISP2 in the opposite compartment. Images are representative of at least 15 GUS-stained seedlings per treatment. Scale bar = 100 μm. Images were acquired using a Zeiss Axio Imager M2 microscope with DIC optics at 20× magnification.

The effect of *S. calvus* (isolate 27) on auxin accumulation was evaluated using the *Arabidopsis DR5::uidA* reporter line. Four-day-old seedlings were co-cultivated with the bacterium under both direct contact and split-plate conditions for 6 days, followed by histochemical GUS staining to visualize auxin-responsive expression patterns in roots (Figure 3). Inoculation with *S. calvus* (isolate 27) significantly enhanced auxin accumulation under all tested conditions. In direct co-cultivation (At-Sc MS) and in split-plate assays, whether the bacterium was grown on MS (At/Sc MS) or on its optimal ISP2 medium (At/Sc ISP2), a significant increase in GUS signal was observed throughout the lateral roots, with stronger signaling at the root tips. In contrast, control seedlings without bacteria displayed only the basal DR5::*uidA* expression pattern, with GUS signals restricted to the root tips. A similar trend occurred in the primary roots, where *S. calvus* induced a markedly stronger GUS signal under both direct and split interactions. In some roots exposed to bacteria, GUS staining was also detected in the vascular tissues (Figure 3). These results indicate that the *S. calvus* isolate 27 promotes greater auxin accumulation in the root system, which may be associated with the increased number of lateral roots observed.

### 2.4. S. calvus Isolate 27 Restores Root Hair Formation in the Arabidopsis rhd6 Mutant Line

**Figure 4:**
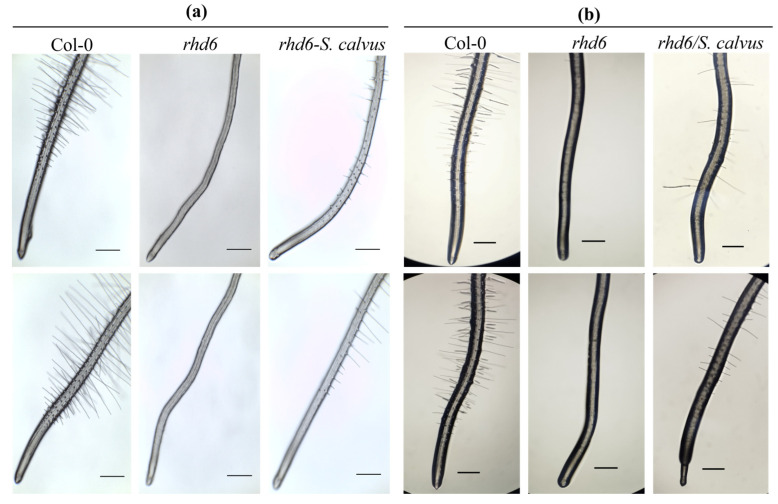
Effect of *Streptomyces calvus* isolate 27 on root hair development in *Arabidopsis rhd6* mutant seedlings. Roots of four-day-old *rhd6* mutant seedlings were inoculated with *S. calvus* spores under direct interaction for 6 days (**a**), or subjected to split interaction (inverted-plate system) for 6 days, in which the spore suspension was applied to a separate plate containing ISP2 medium (**b**). Representative images of *rhd6* roots inoculated with *S. calvus* under direct (*rhd6*-*S. calvus*) and split (*rhd6*/*S. calvus*) interaction conditions are shown. Non-inoculated *A. thaliana* wild-type (Col-0) and *rhd6* roots were included as controls. Scale bar = 500 μm. Images were captured using a stereomicroscope (Motic SMZ-143 series) at 5× magnification and are representative of 12 seedlings per treatment.

The effect of *S. calvus* isolate 27 on the restoration of root hair development in the *rhd6* mutant line was evaluated. Four-day-old seedlings were co-cultivated with the bacterium under both direct and split interaction conditions for 6 days. As shown in Figure 4a, direct inoculation with *S. calvus* partially restored root hair formation in the *rhd6* mutant, whereas non-inoculated mutant seedlings did not develop root hairs. Non-inoculated wild-type (Col-0) seedlings exhibited normal root hair development, as expected. In the split-interaction assay, performed using an inverted-plate system with *S. calvus* grown on ISP2 medium, the bacterium also partially complemented the *rhd6* phenotype (Figure 4b). Although root hair density did not reach the levels observed in the wild-type control, the restoration of root hairs under both direct and split interactions suggests that diffusible and/or volatile compounds produced by the bacterium activate hormonal signaling pathways capable of partially compensating for the genetic defect of the *rhd6* mutant.

### 2.5. Identification of Volatile Organic Compounds Emitted by S. calvus Isolate 27 During Co-Cultivation with Arabidopsis

Volatile organic compound (VOC) profiles emitted by *S. calvus*, both alone and during interaction with *A. thaliana*, were identified using a split-plate system. Six-day-old *Arabidopsis* seedlings were placed on MS medium in the left compartment of Petri dishes, while 30 µL of an *S. calvus* spore suspension (1 × 10^7^ CFU mL^−1^) was inoculated onto ISP2 medium in the right compartment. After 6 days of incubation, VOCs were collected for 1 h and analyzed by GC-MS. After subtracting the VOCs detected in the control media (Supplementary Table S1) and those emitted individually by the plantlets, the compounds specifically produced by *S. calvus*, either alone or during interaction with *Arabidopsis* plantlets, are presented in Table 1. Four VOCs were identified across the two growth conditions analyzed for *S. calvus* isolate 27. After 6 days of growth on ISP2 medium in the absence of plants, isolate 27 produced one alcohol, 1-nonanol (36.41 ± 15.24%), and two sesquiterpenes: selina-3,7(11)-diene (14.19 ± 3.13%) and trans-1,10-dimethyl-trans-9-decalinol (49.38 ± 14.23%). In contrast, during split interaction with *Arabidopsis* plantlets, two sesquiterpenes were detected: trans-1,10-dimethyl-trans-9-decalinol (34.09 ± 4.36%) and methanoazulene (65.91 ± 6.96%). Notably, trans-1,10-dimethyl-trans-9-decalinol was produced under both growth conditions; this compound, commonly known as geosmin, is responsible for the characteristic musty or wet-earth odor typically associated with members of the genus *Streptomyces*.

### 2.6. Growth-Promoting Effects of S. calvus (Isolate 27) on Pot-Grown Arabidopsis and Its Interaction with Trichoderma atroviride

**Figure 5:**
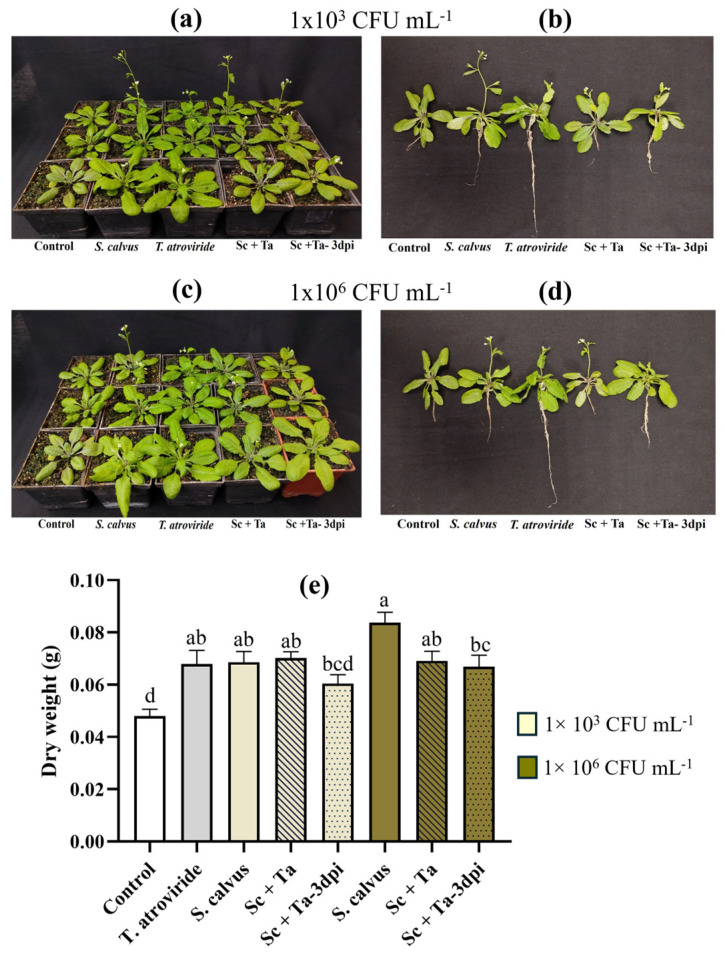
Effect of *Streptomyces calvus* alone or in combination with *Trichoderma atroviride* on the growth of *Arabidopsis* plants grown in soil pots. (**a**–**d**) Representative images of *Arabidopsis* plants under the different treatments at 23 days post-inoculation (dpi). Seven-day-old seedlings were treated with *S. calvus* (Sc) at two concentrations (1 × 10^3^ and 1 × 10^6^ CFU mL^−1^), *T. atroviride* (Ta), co-inoculation of Sc and Ta applied simultaneously (Sc + Ta), Ta applied 3 days after Sc (Sc + Ta- 3dpi), and non-inoculated control plants. (**e**) Plant dry weight at 23 dpi. Data are presented as mean ± SE (*n* = 15). Different letters indicate statistically significant differences among treatments (one-way ANOVA followed by Dunnett’s test for comparisons against the control, *p* ≤ 0.05).

To evaluate the growth-promoting activity of *S. calvus* isolate 27 in pot experiments, seven-day-old *A. thaliana* plants were inoculated with *S. calvus* spores at two concentrations (1 × 10^3^ and 1 × 10^6^ CFU mL^−1^), either alone or in combination with *T. atroviride* (Ta). Integrating *Trichoderma* spp. with root-associated bacteria has been reported to enhance complementary and synergistic effects compared to single-microorganism applications [30]. Accordingly, *T. atroviride*, a well-documented plant growth-promoting fungus [5,6], was selected for co-inoculation experiments. Co-inoculation treatments were performed either simultaneously or sequentially, with *T. atroviride* applied three days after the initial bacterial inoculation. At 23 days post-inoculation (dpi), all treatments, including *S. calvus* alone, *T. atroviride* alone, and their combinations, produced more vigorous plants, characterized by increased rosette size and greater inflorescence development compared to the uninoculated control (Figure 5a–d). Plants treated with *S. calvus* at the highest concentration (1 × 10^6^ CFU mL^−1^) exhibited the most pronounced inflorescence development, whereas maximal root elongation was observed in plants inoculated exclusively with *T. atroviride*. Analysis of dry weight revealed that plants treated with *S. calvus* at 1 × 10^6^ CFU mL^−1^ showed the highest biomass accumulation, exceeding all other treatments. The lower bacterial concentration (1 × 10^3^ CFU mL^−1^) resulted in dry weight values comparable to those observed in plants inoculated solely with *T. atroviride* or in simultaneous co-inoculation treatments (Sc + Ta). In contrast, sequential inoculation, in which fungal application was delayed by three days, resulted in the lowest dry weight value among the treatments. This reduction was particularly pronounced at the lower bacterial concentration (1 × 10^3^ CFU mL^−1^), where biomass accumulation was statistically similar to the uninoculated control, indicating that the sequential strategy abolished the growth-promoting effect only under this condition. At the higher bacterial concentration (1 × 10^6^ CFU mL^−1^), sequential inoculation with *T. atroviride* still resulted in significantly greater biomass compared with the control. Collectively, these findings indicate that *S. calvus* isolate 27 functions as an effective plant growth-promoting agent in *A. thaliana* under soil conditions, demonstrating efficacy both as a single inoculant and when simultaneously co-inoculated with *T. atroviride*.

### 2.7. Inoculation with S. calvus Isolate 27 Promotes Growth and Development in Tomato Plants

**Figure 6:**
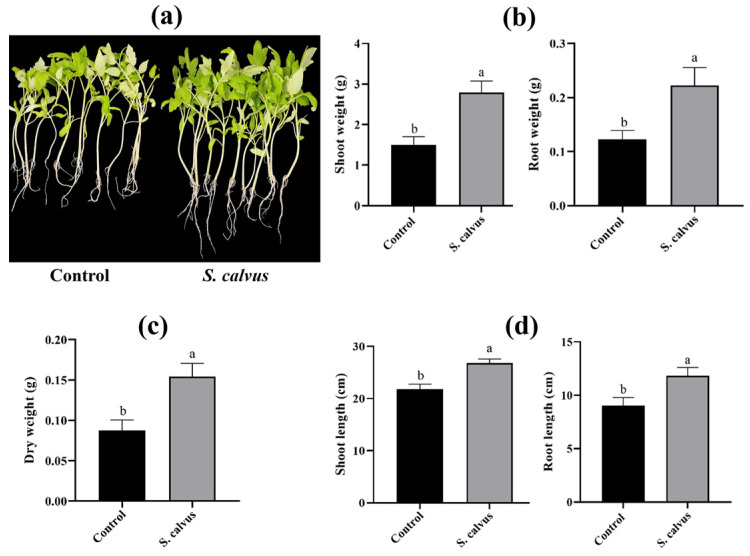
Effect of inoculation with *S. calvus* isolate 27 on the growth of tomato plants grown in pots with substrate. (**a**) Images of tomato plants inoculated at the seed stage with *S. calvus* isolate 27 at 28 days post-inoculation (dpi). (**b**) Fresh weight of shoots and roots. (**c**) Dry weight of whole plants. (**d**) Shoot and root length. All measurements were taken at 28 dpi. Data are expressed as mean ± SE (*n* = 12). Different letters indicate statistically significant differences between treatments (Student’s *t*-test, *p* ≤ 0.05).

To evaluate the growth-promoting effects of *S. calvus* isolate 27 on tomato plants, seeds were sown and inoculated with spores at 1 × 10^6^ CFU mL^−1^ and compared with non-inoculated controls. After four weeks of interaction, inoculated plants exhibited enhanced growth and a more developed phenotype relative to the control (Figure 6a). Fresh weight of shoots and roots increased more than 1.8-fold compared with non-inoculated plants, while total dry weight was 1.7-fold (Figure 6b,c). In addition to biomass accumulation, isolate 27 promoted a more developed plant phenotype, increasing shoot and root length by approximately 1.2- and 1.3-fold, respectively. Collectively, these results indicate that *S. calvus* isolate 27 modulates root architecture and consequently enhances shoot development in tomato plants.

## 3. Discussion

In this study, we aimed to characterize an actinobacterium (isolate 27) isolated from the rhizospheric soil of melon plants and evaluate its potential as a plant growth-promoting microorganism (PGPM). Although research on actinobacteria has traditionally focused on their biotechnological applications in antibiotic production, there is a growing interest in understanding their beneficial interactions with plants. Molecular and morphological data support the assignment of isolate 27 to the *S. calvus* species. The 16S rRNA sequence of isolate 27 exhibited high similarity to *S. calvus* strains. Consistently, phylogenetic analysis of the 16S rRNA gene sequence placed isolate 27 within the *S. calvus* clade.

Isolate 27 exhibited the characteristic morphology and traits of the genus *Streptomyces*, including branched filamentous mycelium, aerial mycelium formation, and spore production [29]. Consistent with these features, isolate 27 emitted the typical earthy odor associated with geosmin, a volatile organic compound synthesized by most species within this genus [31]. The sand-yellow pigmentation of the substrate mycelium of isolate 27 is consistent with previously described phenotypic characteristics of *S. calvus* grown on ISP2 medium [29]. Notably, isolate 27 exhibited the “bald” phenotype characteristic of this species, marked by deficient aerial mycelium and spore formation on most standard media except ISP2 [29]. This phenotype has been attributed to a point mutation in the *bldA* gene, which encodes the Leu-tRNA^UUA required for the translation of key regulatory genes involved in morphological development. The complete restoration of sporulation through genetic complementation with a functional copy of *bldA* confirms its central role in *Streptomyces* morphogenesis [32]. However, this mutation can be suppressed under specific nutritional and environmental conditions provided by the culture medium, resulting in the recovery of morphological differentiation through the expression of genes involved in the formation of aerial mycelium [29].

Thus, the molecular, morphological, and colony characteristics observed in this study, including the 16S rRNA gene sequence analysis, well-developed aerial and substrate mycelia, spiny-surfaced spores, and the absence of diffusible pigments on ISP2 medium, are consistent with the established taxonomic description of *S. calvus*.

Since isolate 27 was obtained from melon rhizospheric soil, this suggests potential plant-beneficial interactions, as diverse *Streptomyces* species have been known to promote plant growth [33]. The growth-promoting effects observed following inoculation with *S. calvus* isolate 27, under both direct contact and split interaction *in vitro* conditions, demonstrate the capacity of this isolate to stimulate biomass accumulation and modulate root system architecture in *A. thaliana* seedlings. The consistent increases in fresh weight and lateral root formation under both interaction types suggest that a combination of diffusible metabolites and/or volatile organic compounds (VOCs) emitted by isolate 27 contributes to these growth-enhancing effects. Notably, the most pronounced plant growth effects were observed when the actinobacterium was grown in ISP2 medium in the adjacent compartment of the split-plate system. This suggests that optimal growth conditions for isolate 27, such as those provided by ISP2, may enhance the production of VOCs that are particularly effective at promoting plant growth. A similar effect was reported by González-Pérez et al. [5], who observed that VOCs emitted by *Trichoderma atroviride* and *T. virens* promoted increased growth of *Arabidopsis* plantlets when the fungi were grown on the optimal medium PDA, compared with MS medium, highlighting the influence of nutrient availability on the metabolic profile associated with VOC-mediated plant growth promotion.

To explore the mechanisms underlying plant growth promotion by isolate 27, auxin accumulation in *A. thaliana* roots was examined using the *DR5::uidA* reporter line. Isolate 27 induced a marked increase in auxin accumulation, as evidenced by enhanced GUS signals under both direct and long-distance interaction conditions. The intensified signal in primary and lateral root tips suggests that this actinobacterium produces diffusible metabolites and/or VOCs that stimulate auxin biosynthesis and/or redistribution. These findings suggest that the increase in lateral root formation could be associated with the modulation of auxin distribution in the roots. Moreover, *S. calvus* isolate 27 partially restored root hair formation in the *Arabidopsis rhd6* mutant, which is defective in early root hair initiation [34]. Since the *rhd6* phenotype can be rescued by exogenous auxin or ethylene, the recovery observed under both direct and long-distance interactions suggests that isolate 27 produces diffusible and/or volatile molecules capable of activating hormone signaling pathways required for root hair initiation. Together, these results support a hormone-mediated mechanism underlying root developmental modulation by *S. calvus* isolate 27.

Volatile organic compounds (VOCs) emitted by beneficial microbes are increasingly recognized for their positive effects on plant physiology and development. These low-molecular-weight compounds function as key chemical signals mediating plant-microbe interactions within the rhizosphere [35,36]. Notably, microbial VOCs can promote plant growth, modulate hormonal signaling pathways, and activate defense responses against biotic and abiotic stresses [5,6,37].

Despite advances in VOC profiling of beneficial microbes, many microorganisms with biotechnological potential remain unexplored, including *Streptomyces* species capable of producing bioactive volatiles. In this study, the volatile profile of *S. calvus* isolate 27 was characterized during interaction with *A. thaliana*, as well as when the actinobacterium was grown in the absence of the plant. When grown solely on ISP2 medium, isolate 27 produced three main compounds: the sesquiterpenes selina-3,7(11)-diene and geosmin, and the alcohol 1-nonanol. In contrast, plant interaction markedly modified the VOC profile; geosmin persisted but decreased in relative abundance, methanoazulene emerged as a new sesquiterpene, and the other metabolites were no longer detected. This shift suggests that the presence of the plant may trigger a reprogramming of bacterial secondary metabolism.

Geosmin, consistently detected under both conditions in this study, is a characteristic and widely conserved metabolite of Actinobacteria. It has been proposed to play a key role in the ecological interactions of *Streptomyces*, and its biosynthesis is closely associated with the developmental cycle of these bacteria, particularly with the onset of sporulation [38,39]. On the other hand, the absence of 1-nonanol and selina-3,7(11)-diene during plant interaction suggests that these volatiles are preferentially produced under free-living conditions. Notably, 1-nonanol has been linked to antimicrobial activity [40]. Although selina-3,7(11)-diene lacks functional characterization in its pure form, it has been identified in plant extracts with reported antimicrobial properties [41]. This compound is also produced by *Streptomyces* sp. (isolate ACTB-77) in axenic culture [42].

Methanoazulene, detected during the split interaction between *S. calvus* and *Arabidopsis*, has also been reported in the VOC profiles of other *Streptomyces* species, such as *S. rochei*, where it was identified among the volatile compounds produced during tripartite interactions with the fungal pathogens *Fusarium moniliforme* and *Curvularia lunata* [43]. In addition, VOCs from *S. rochei* not only exhibited antifungal activity but also promoted sorghum growth, increasing shoot length and biomass, supporting their dual role in pathogen suppression and plant growth promotion. Although the VOCs detected in *S. calvus* isolate 27 were consistently present under conditions in which plant growth promotion was observed in split-plate assays, no studies have yet demonstrated their direct involvement. Therefore, future studies evaluating the effects of these individual VOCs and their mixtures on plants will be essential to further elucidate their role in plant growth promotion. Notably, recent evidence demonstrates that *Streptomyces* species can regulate plant growth and stress tolerance through volatile-mediated aerial signaling. Qin et al. (2024) showed that *S. setonii* WY228 promotes plant growth and enhances salt stress tolerance via VOCs, demonstrating that actinobacteria can modulate plant physiology in the absence of physical contact [44].

It is worth noting that *S. calvus* isolate 27 had a positive impact on the growth of pot-grown *A. thaliana* at both concentrations evaluated. However, the higher concentration promoted greater vegetative development and increased dry weight accumulation in *Arabidopsis* plants. The effect observed at the higher concentration of isolate 27 may be attributed to a greater bacterial density, which could enhance the production of growth-promoting metabolites as well as improve root colonization efficiency. Furthermore, when the actinobacterium was simultaneously co-inoculated with *T. atroviride*, plants exhibited greater development compared to the uninoculated control. The use of microbial consortia has increased in recent years, as numerous studies have reported increased plant growth, based on the understanding that microorganisms naturally coexist in complex communities and interact in a coordinated manner with plants [30]. Additionally, it has been demonstrated that consortia of *T. atroviride* with different plant growth-promoting bacteria increased both pathogen inhibition and growth promotion in *Arabidopsis*, effects associated with the induction of key fungal effector genes such as *epl1* and *tatrx2* [45]. In this context, it has been reported that overexpression of the *epl1* gene from *T. atroviride* in *Arabidopsis* resulted in plants with enhanced growth and increased resistance against *Botrytis cinerea* and *Pseudomonas syringae*, confirming the role of the elicitor from *Trichoderma* in plant growth and defense responses [46]. These findings suggest that the interaction between bacteria and fungi within a consortium may involve functional coordination that enhances the activation of plant defense and growth mechanisms. In contrast, sequential co-inoculation, in which the bacterium was applied first and the fungus three days later, resulted in the lowest positive impact on plant growth and development compared with the other treatments evaluated. The co-inoculation of *T. harzianum* and the actinobacterium *S. microflavus* in tomato resulted in a smaller root system compared to plants inoculated with individual strains [47]. Transcriptomic analysis revealed enhanced activation of defense-related genes, suggesting that plants reallocated part of their energy from development toward defense activation when co-inoculated with these biological control agents. Together, these findings highlight the complexity of microbial consortia and the importance of analyzing inoculation strategies and microbial dynamics when designing efficient bioinoculants.

A similar effect to that observed in *Arabidopsis* was obtained when *S. calvus* isolate 27 was inoculated onto tomato seeds in a soil-based substrate. The results indicate that this actinobacterium significantly promotes plant growth, resulting in larger and more vigorous individuals, as evidenced by a significant increase in total biomass as well as shoot and root length. The enhanced development of the root system suggests an improved capacity for water and nutrient uptake, which likely contributes to the greater vigor observed in the inoculated plants. Overall, these data highlight the potential of *S. calvus* isolate 27 as a potent biostimulant to enhance the growth of both *Arabidopsis* and tomato plants.

To the best of our knowledge, there are no previous reports describing plant growth promotion by *S. calvus*, making this study the first evidence of its potential as a plant growth-promoting (PGP) actinobacterium. Nevertheless, related information is available for closely related species. For instance, *S. asterosporus* has recently been proposed as a later heterotypic synonym of *S. calvus* [48]. Notably, strain SNL2 of *S. asterosporus* promotes tomato growth through the production of auxins and siderophores [49]. Other species of *Streptomyces* have also been described as plant growth promoters, underscoring the broad biostimulant potential within this genus. Rhizospheric actinomycetes were isolated from wheat and tomato fields, and their effects were evaluated on wheat seedlings. Among them, *S. nobilis, S. djakartensis,* and *S. enissocaesilis* showed the strongest growth-promoting effects, enhancing shoot and root length, biomass accumulation, and the number of leaves and roots compared to uninoculated controls [50]. Endophytic actinobacteria isolated from roots of *Opuntia ficus-indica* with plant growth-promoting (PGP) activity in wheat have been identified [51]. The isolates exhibited multiple PGP traits, including siderophore production, nitrogen fixation, auxin synthesis, and ACC deaminase activity, which were associated with significant improvements in seedling development. Among the most effective strains, *S. tuirus* increased the number of lateral roots, *S. levis* promoted the greatest seedling and root lengths, and *S. radiopugnans* induced the highest shoot growth. Inoculated seedlings also maintained a healthy green phenotype even in the absence of an external water supply. These findings show that *Streptomyces* strains isolated from abiotic stress-tolerant species such as cactus can serve as effective bioinoculants for crops under stress conditions.

## 4. Materials and Methods

### 4.1. Isolation and Characterization of Actinobacterial Isolate 27

The actinobacterial isolate 27 was isolated from the rhizospheric soil of melon plants (*Cucumis melo* L.) in Paila, a locality within the municipality of Parras de la Fuente, Coahuila, Mexico (25°39′28.25″ N; 101°53′51.74″ W). The bacterium was grown on ISP2 medium to induce sporulation at 28 °C for two weeks. ISP2 medium (International *Streptomyces* Project-2) contained yeast extract (4 g L^−1^, MCD Lab, Tultitlán de Mariano Escobedo, México), malt extract (10 g L^−1^, MCD Lab, Tultitlán de Mariano Escobedo, México), glucose (4 g L^−1^, Sigma Aldrich, St. Louis, MO, USA), and agar (15 g L^−1^, Sigma Aldrich, St. Louis, MO, USA). The isolate was also cultured on ISP3 medium (International *Streptomyces* Project-3) composed of rolled oats (20 g L^−1^), agar (18 g L^−1^), and 1 mL of a trace salts solution (0.1 g FeSO_4_·7H_2_O, Fermont, Monterrey, México, 0.1 g MnCl_2_·4H_2_O, Sigma Aldrich, St. Louis, MO, USA, and 0.1 g ZnSO_4_·7H_2_O, Fermont, Monterrey, México, in 100 mL distilled H_2_O), adjusted to pH 7.2. For morphological characterization, isolate 27 was cultured on ISP2 and ISP3 media for two weeks to evaluate the development of aerial and substrate mycelia. The microscopic structures of hyphae and spores were examined from a four-week-old culture grown on ISP2 medium. For scanning electron microscopy (SEM), samples were processed and observed using an ESEM FEI QUANTA 250 microscope (FEI, Model 1027641, Brno, Czech Republic); before observation, they were gold-coated to ensure conductivity. SEM observations focused on spore chain morphology and mycelial branching. For Gram staining, bacterial cultures were grown in nutrient broth. The staining procedure was performed using the HYCEL Gram Staining Kit (cat. 541-EQ, HYCEL, Zapopan, México) according to the manufacturer’s instructions. This method is based on crystal violet staining. Finally, the samples were observed under a light microscope at 100× magnification using immersion oil.

### 4.2. Molecular Identification of Actinobacterial Isolate 27

Molecular identification was performed by PCR amplification of the 16S rRNA gene using universal primers (63F and 1387R) [52], targeting the V2-V8 regions, generating an amplicon of 1.3 kb. Genomic DNA was extracted following the protocol of Wilson [53], with minor modifications, specifically in the volumes of the reagents used. PCR conditions consisted of an initial denaturation at 95 °C for 3 min, followed by 35 cycles of 95 °C for 30 s, 60 °C for 30 s, and 72 °C for 1 min, with a final extension at 72 °C for 7 min. The PCR product was purified using the Wizard^®^ SV Gel and PCR Clean-Up System (Promega, Madison, WI, USA) and sequenced. The sequence was deposited in GenBank (accession number PX674612) and compared with reference sequences using BLAST [54]. A Neighbor-Joining phylogenetic tree based on the 16S rRNA gene was constructed using MEGA11 [55], with *Nocardia casuarinae* as the outgroup. *S. calvus* isolate 27 was preserved in liquid ISP2 medium at −80 °C.

### 4.3. Plant Material and Growth Conditions

Seeds of *A. thaliana* (ecotype Col-0), the *DR5::uidA* reporter line, and the *rhd6* mutant were used in this study. Seeds were surface-sterilized in a 3% (*v*/*v*) sodium hypochlorite solution for 10 min, rinsed four times with sterile distilled water, and stratified at 4 °C for 2 days. After sterilization, seeds were sown on Petri dishes containing 0.2× Murashige and Skoog (MS, PhytoTech Labs, Lenexa, KS, USA) medium supplemented with vitamins, 1% (*w*/*v*) sucrose, and 1% (*w*/*v*) agar, adjusted to pH 5.7. Plates were incubated in a growth chamber under a 16 h light (120 μmol m^−2^ s^−1^)/8 h dark photoperiod at 22 ± 1 °C for the duration specified in each experiment.

### 4.4. In Vitro Interaction Assays Between S. calvus and A. thaliana

Co-culture assays between *S. calvus* (isolate 27) and *A. thaliana* Col-0 were performed for 7 days. Six-day-old seedlings were placed on one side of the divided Petri dishes (9.0 × 1.5 cm) containing 0.2× MS medium. For inoculation, 30 µL of *S. calvus* spore suspension (1 × 10^7^ CFU mL^−1^) was applied either to the same side (direct interaction) or to the opposite side (split interaction). Six replicate plates were used, each containing three seedlings. Five conditions were evaluated: (i) MS without bacteria (control At/MS); (ii) direct inoculation on MS (At-Sc MS); (iii) split plates with MS on both sides and bacteria on the opposite side (At/Sc MS); (iv) MS with ISP2 on the opposite side without bacteria (control At/ISP2); and (v) MS with bacteria placed on ISP2 on the opposite side (At/Sc ISP2). After 7 dpi under controlled conditions (Section 4.3), plantlets were analyzed phenotypically. Plates were photographed individually. For fresh weight determination, three plantlets per plate were pooled (*n* = 6). Lateral roots were counted from digital images (*n* = 18). The assay was repeated in three independent experiments with similar results.

### 4.5. Histochemical Detection of GUS Activity in the DR5::uidA Reporter Line During S. calvus Interaction

Four-day-old *DR5::uidA* seedlings were inoculated with 30 µL of *S. calvus* spore suspension (1 × 10^7^ CFU mL^−1^) and subjected to the following conditions: (i) non-inoculated control seedlings grown on MS; (ii) direct interaction with the bacterium on MS; (iii) split interaction with seedlings grown on MS and the bacterium cultured on MS in the opposite compartment; and (iv) split interaction with seedlings grown on MS and the bacterium cultured on ISP2 in the opposite compartment. After 6 days of interaction, seedlings were subjected to GUS histochemical staining by incubation for 16 h at 37 °C in GUS reaction buffer containing 0.5 mg mL^−1^ of 5-bromo-4-chloro-3-indolyl-β-D-glucuronide (PhytoTech Labs, Lenexa, KS, USA) in 100 mM sodium phosphate buffer, pH 7 (Sigma Aldrich, St. Louis, MO, USA), according to Jefferson [56]. Seedlings were subsequently cleared as described by Malamy and Benfey [57]. For each condition, at least 15 seedlings were analyzed (five plates with three seedlings each). Root images were acquired as described by González Pérez et al. [5] using a Zeiss Axio Imager M2 microscope (Göttingen, Germany) equipped with DIC optics (10× objective) and processed with ZEN2 software (blue edition, 2.0.14283.302). The experiment was independently repeated at least three times with similar results.

### 4.6. Interaction of S. calvus Isolate 27 with Arabidopsis rhd6 Mutant Seedlings

Four-day-old *rhd6* mutant seedlings were subjected to direct and split interaction with *S. calvus* isolate 27. For direct interaction, 5 µL of a 2 × 10^8^ CFU mL^−1^ *S. calvus* spore suspension was applied to the root tip of each seedling. For split interaction, an inverted-plate system was employed, in which 30 µL of an *S. calvus* spore suspension (1 × 10^7^ CFU mL^−1^) was streaked in a line onto ISP2 medium and grown for two days at 28 °C before interaction. For both interaction systems, seedlings were maintained for 6 days. At 10 days of age, roots were photographed using a stereomicroscope (Motic SMZ-143, San Antonio, TX, USA). Twelve seedlings per treatment were analyzed. The experiment was independently repeated at least twice with similar results.

### 4.7. Fungal Isolate and Inoculum Preparation

*Trichoderma atroviride* (strain IMI 206040) was grown on potato dextrose agar (PDA; BD Difco™ Laboratories, Sparks, MD, USA) for eight days at 28 °C. Spores were collected in sterile water and quantified at 40× using a Neubauer chamber and a Motic BA-300 microscope (Motic^®^, San Antonio, TX, USA). For each treatment, 1 mL per pot of a suspension containing 2.5 × 10^6^ spores mL^−1^ was applied.

### 4.8. Inoculation Assays of S. calvus (Isolate 27) and Co-Inoculation with T. atroviride in Pot-Grown Arabidopsis

Seven-day-old *A. thaliana* (Col-0) seedlings were transplanted into pots (6 × 7 cm, approximately 200 mL) containing sterile substrate (Sunshine Mix^®^ #3, vermiculite, and perlite; 3:1:1). After three days of acclimation, 15 replicates per treatment (*n* = 15) were inoculated by applying 1 mL of *S. calvus* spore suspension to the base of the stem. Treatments included: *S. calvus* (Sc) at 1 × 10^3^ and 1 × 10^6^ CFU mL^−1^; *T. atroviride* (Ta) alone; simultaneous co-inoculation (Sc + Ta); sequential co-inoculation (with Ta applied 3 days after Sc); and a non-inoculated control. In consortium treatments, 0.5 mL of each microorganism was applied. Plants were maintained under controlled conditions (16/8 h light/dark photoperiod, 120 µmol m^−2^ s^−1^, 22 ± 1 °C) and watered every three days. At 23 days post-inoculation (dpi), plants were harvested for photographic documentation and dry weight determination by drying in an oven (BINDER ED 400-230V, Tuttlingen, Germany) at 60 °C for 72 h.

### 4.9. Tomato Growth Assay Following Inoculation with S. calvus

Tomato seeds (*Solanum lycopersicum* L., cv. Rancho Grande) were surface-sterilized with 3% (*v*/*v*) sodium hypochlorite for disinfection, rinsed four times with sterile distilled water, and stored at 4 °C for 48 h. Seeds were sown in sterile substrate (Sunshine Mix^®^ #3: vermiculite: perlite, 3:1:1) in 3 L pots (*n* = 12). At sowing, each seed received 1 mL of *S. calvus* isolate 27 spore suspension (1 × 10^6^ CFU mL^−1^), while non-inoculated plants were used as controls. Plants were grown in a controlled growth chamber under a 16 h light/8 h dark photoperiod at 22 ± 1 °C and irrigated with water every three days. After 28 days, plants were harvested, photographed, and growth parameters, including shoot and root length, as well as fresh and dry weight, were measured. Experiments were performed at least twice with similar results.

### 4.10. Collection and Analysis of S. calvus Isolate 27 Volatile Compounds

Volatile compounds emitted by *S. calvus* isolate 27 were analyzed using a split-interaction system. Four conditions were evaluated: (1) ISP2 medium alone, (2) uninoculated *Arabidopsis* seedlings grown on MS medium, (3) isolate 27 grown on ISP2 without plants, and (4) the interaction between *S. calvus* on ISP2 and *Arabidopsis* seedlings on MS medium.

Before the experiment, a small hole (1.5 mm in diameter) was made in each Petri dish to allow insertion of the solid-phase microextraction (SPME) fiber. Three plates were analyzed for each condition (*n* = 3). Three 6-day-old *Arabidopsis* seedlings were placed on the side of the plate containing the hole, while 30 µL of *S. calvus* spore suspension (1 × 10^7^ CFU mL^−1^) was inoculated on the opposite side. Plates were sealed with Parafilm to prevent loss of volatile compounds and incubated in a growth chamber under a 16 h light (120 μmol m^−2^s^−1^)/8 h dark photoperiod at 22 ± 1 °C for six days before analysis. Volatile compounds were collected using a polydimethylsiloxane/divinylbenzene SPME fiber and analyzed by GC-MS (Agilent 7890B/5977A, Agilent Technologies, Santa Clara, CA, USA) equipped with an HP-Innowax capillary column. Fibers were exposed for 1 h and compounds were desorbed at 200 °C in splitless mode. The oven program was 40 °C (10 min) to 180 °C at 3 °C min^−1^ (10 min hold) using helium as carrier gas (1.5 mL min^−1^). Compounds were identified using the Wiley10Nist11 database and linear retention indices calculated with C6-C25 n-alkanes. Relative abundances were estimated from peak areas and expressed as percentages, as previously described by González-Pérez et al. [5].

### 4.11. Statistical Analysis

Growth parameters in *Arabidopsis* and tomato plants, including fresh and dry weight, shoot and root length, and the number of lateral roots, were analyzed using one-way analysis of variance (ANOVA) or Student’s *t*-test, as appropriate. When more than two groups were compared, Dunnett’s multiple comparison test was applied with a significance level of *p* ≤ 0.05. All statistical analyses were performed using GraphPad Prism version 8.0 (GraphPad Software, Inc., San Diego, CA, USA). Data are presented as mean ± SE, and statistically significant differences are indicated by different letters.

## 5. Conclusions

In this study, isolate 27 was identified as *Streptomyces calvus* and characterized as a plant growth-promoting microorganism. Its biostimulant activity was initially demonstrated *in vitro* using *Arabidopsis thaliana* seedlings and subsequently validated in pot experiments with substrate in both *A. thaliana* and tomato, where it induced significant increases in biomass as well as enhanced shoot and root development. The growth-promoting mechanisms of isolate 27 involve modulation of plant hormonal pathways, specifically through the induction of auxin accumulation, as evidenced by the increased activity of the *DR5::uidA* reporter and the rescue of the *rhd6* mutant phenotype. Volatile organic compounds produced by isolate 27 were detected in split-plate systems associated with enhanced plant growth, although their specific functional roles remain to be elucidated. Future transcriptomic and metabolomic analyses will be essential to elucidate plant metabolic pathways induced by *S. calvus*-derived signals. Overall, isolate 27 of *S. calvus* represents a promising bioinoculant for sustainable agriculture, with consistent performance as both a single inoculant and a component of microbial consortia, supporting its potential to enhance crop productivity (*e.g.*, tomato) while reducing dependence on chemical fertilizers and contributing to more sustainable and resilient agroecosystems.

## Figures and Tables

**Table 1:** Relative abundance (%) of volatile organic compounds (VOCs) emitted by *Streptomyces calvus* isolate 27 in a split-plate system under two conditions: (i) MS without plants in the left compartment and isolate 27 grown on ISP2 in the right compartment; and (ii) MS with plants in the left compartment and isolate 27 grown on ISP2 in the right compartment. Values are presented as mean ± SE from three biological replicates, n.d.: not detected.

No.	Class	Compound	*S. calvus* Without Plant Interaction (%)	*S. calvus* Plant Interaction (%)
1	Alcohol	1-Nonanol	36.41 ± 15.24	n.d.
2	Sesquiterpene	Selina-3,7(11)-diene	14.19 ± 3.13	n.d.
3	Sesquiterpene	Trans-1,10-dimethyl-trans-9-decalinol	49.38 ± 14.23	34.09 ± 4.36
4	Sesquiterpene	Methanoazulene	n.d.	65.91 ± 6.96

## Data Availability

The original contributions presented in this study are included in the article/Supplementary Material. Further inquiries can be directed to the corresponding author.

## Supplementary Materials

The following supporting information can be downloaded at: https://www.mdpi.com/article/10.3390/plants15091315/s1, Figure S1: Phylogenetic tree based on 16S rRNA gene sequences of *Streptomyces calvus* strain 27 (accession number PX674612.1) isolated in the present study and other *Streptomyces* species obtained from GenBank. The tree was constructed using the Neighbor-Joining method. *Nocardia casuarinae* BMG51109 (KF924767) was used as an outgroup. Values at the branches indicate bootstrap support based on 1000 replicates; Table S1: Volatile organic compounds (VOCs) emitted in ISP2 medium.
