# Supplementary material for: Streptomyces calvus Isolate 27 Promotes Plant Growth Through Hormone Accumulation and Bioactive Compounds
Source: Plants (Basel). 2026 Apr 25;15(9):1315. doi: 10.3390/plants15091315 (PMC13165174; doi:10.3390/plants15091315)
Supplement: Supplementary file 1 [file plants-15-01315-s001.zip › plants-4218454-supplementary.pdf]

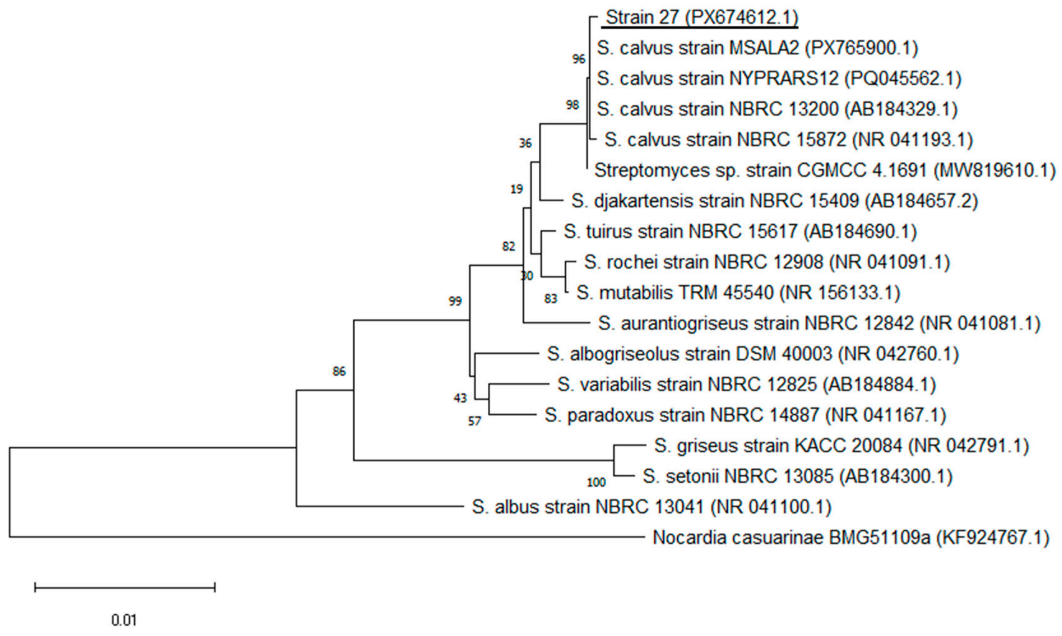

Supplementary Figure S1. Phylogenetic tree based on 16S rRNA gene sequences of *Streptomyces calvus* strain 27 (accession number PX674612.1) isolated in the present study and other *Streptomyces* species obtained from GenBank. The tree was constructed using the Neighbor-Joining method. *Nocardia casuarinae* BMG51109 (KF924767) was used as an outgroup. Values at the branches indicate bootstrap support based on 1000 replicates.

Supplementary Table S1. Volatile organic compounds (VOCs) produced in ISP2 medium

**ISP2 medium**
Nonanal
Propanal
Furfural
1-hexanol, 2-ethyl
Benzaldehyde
1-phenyl-ethanone
Benzaldehyde, 2-hydroxy
Thiophene, 2,4-dimethyl
Benzenemethanol
1-dodecanol
Phenol 3,5-Dioctoxyphenol
4-Quinolinecarboxaldehyde
